# What an *Escherichia coli* Mutant Can Teach Us About the Antibacterial Effect of Chlorophyllin

**DOI:** 10.3390/microorganisms7020059

**Published:** 2019-02-22

**Authors:** Marcus Krüger, Peter Richter, Sebastian M. Strauch, Adeel Nasir, Andreas Burkovski, Camila A. Antunes, Tina Meißgeier, Eberhard Schlücker, Stefan Schwab, Michael Lebert

**Affiliations:** 1 Clinic for Plastic, Aesthetic and Hand Surgery, Otto von Guericke University Magdeburg, Leipziger Str. 44, 39120 Magdeburg, Germany; 2 Cell Biology Division: Gravitational Biology Group, Department of Biology, Friedrich-Alexander University Erlangen-Nuremberg, Staudtstraße 5, 91058 Erlangen, Germany; peter.richter@fau.de (P.R.); sebastian.m.strauch@fau.de (S.M.S.); adeel.nasir@fau.de (A.N.); tina.meissgeier@fau.de (T.M.); michael.lebert@fau.de (M.L.); 3 Microbiology Division, Department of Biology, Friedrich-Alexander University Erlangen-Nuremberg, Staudtstraße 5, 91058 Erlangen, Germany; andreas.burkovski@fau.de (A.B.); camila.a.azevedo@fau.de (C.A.A.); 4 Institute of Process Machinery and Systems Engineering (iPAT), Friedrich-Alexander University Erlangen-Nuremberg, Cauerstraße 4, 91058 Erlangen, Germany; sl@ipat.fau.de (E.S.); sw@ipat.fau.de (S.S.)

**Keywords:** chlorophyll, photosensitization, antimicrobial photodynamic therapy, aPDT, alternative antibiotics

## Abstract

Due to the increasing development of antibiotic resistances in recent years, scientists search intensely for new methods to control bacteria. Photodynamic treatment with porphyrins such as chlorophyll derivatives is one of the most promising methods to handle bacterial infestation, but their use is dependent on illumination and they seem to be more effective against Gram-positive bacteria than against Gram-negatives. In this study, we tested chlorophyllin against three bacterial model strains, the Gram-positive *Bacillus subtilis* 168, the Gram-negative *Escherichia coli* DH5α and *E. coli* strain NR698 which has a deficient outer membrane, simulating a Gram-negative “without” its outer membrane. Illuminated with a standardized light intensity of 12 mW/cm^2^, *B. subtilis* showed high sensitivity already at low chlorophyllin concentrations (≤10^5^ cfu/mL: ≤0.1 mg/L, 10^6^–10^8^ cfu/mL: 0.5 mg/L), whereas *E. coli* DH5α was less sensitive (≤10^5^ cfu/mL: 2.5 mg/L, 10^6^ cfu/mL: 5 mg/L, 10^7^–10^8^ cfu/mL: ineffective at ≤25 mg/L chlorophyllin). *E. coli* NR698 was almost as sensitive as *B. subtilis* against chlorophyllin, pointing out that the outer membrane plays a significant role in protection against photodynamic chlorophyllin impacts. Interestingly, *E. coli* NR698 and *B. subtilis* can also be inactivated by chlorophyllin in darkness, indicating a second, light-independent mode of action. Thus, chlorophyllin seems to be more than a photosensitizer, and a promising substance for the control of bacteria, which deserves further investigation.

## 1. Introduction

The enormous proliferation of bacteria together with efficient mechanisms for horizontal gene transfer such as conjugation enable fast adaptation to arduous environmental conditions. In the competition for food sources, antibiotic production by some microorganisms appears to be important for survival. Antibiotics can be seen as significant impediment for bacterial growth. The use of antibiotics to destroy harmful microorganisms represented fundamental progress in medicine, resulting in the treatment of previously incurable diseases [1,2]. However, if a certain antibiotic is constantly present in the environment it is only a matter of time until mutations and gene transfer processes will select bacterial strains, which develop resistance and transmit this resistance genes e.g., via conjugation to other possibly harmful bacteria [3]. Boosted by a careless use of antibiotics in medicine [4,5], livestock breeding [6,7], or aquaculture [8], resistances against almost all known antibiotics have developed. In particular, the use of antibiotics in sub-inhibitory concentrations in animal farms is regarded as an important factor of resistance development in bacteria [9]. Certain bacteria such as *Staphylococcus aureus* are very efficient recipients of R(esistance)-plasmids, making them immune against a complete set of antibiotics (multidrug resistance; MDR) [10]. This is an enormous problem in hospitals, especially in intensive care units, which are hot spots of resistance generation, because bacteria are in almost constant contact with various antibiotics here. Due to infections with mostly MDR hospital germs, in the United States every year nearly two million people were infected from which about 99,000 die [11]. Also in the European Union (EU), antimicrobial resistance causes 25,000 deaths per year [12]. A recent EU-wide statistic found the annual burden of these infections is similar to the combined burden of influenza, tuberculosis, and HIV [13]. For long time carbapenems, broad spectrum agents with high bactericidal activity, were considered as “last-resort” antimicrobials in therapy of infections caused by MDR pathogens [14]. Nevertheless, first carbapenem-resistant *Enterobacteriaceae* such as some *Klebsiella pneumoniae* strains have emerged as a major threat during recent years [15]. In addition, most recently, a previously unrecognized spread of nearly pan-drug-resistant, hospital-adapted lineages of *Staphylococcus epidermidis* was uncovered [16]. Due to the lack of active antimicrobials, infections can reach mortality rates of 23 to 75% [17]. The number of deaths caused by antimicrobial resistance is expected to increase to 10 million deaths a year worldwide by 2050 [18]. Thus, it is no wonder that the growing prevalence of pathogens resistant to most or even all currently available antimicrobial agents heralds the potential risk of an upcoming “post-antibiotic era” for many scientists [19,20,21,22,23,24].

To cope with the adaptation of bacteria to a remedy, one strategy is to identify new antibiotics with new target structures. For example, teixobactin was recently isolated from a soil bacterium. So far, no resistance of certain pathogenic bacteria strains against teixobactin could be induced [25]. Another promising approach is the light-dependent inactivation of bacteria via antimicrobial photodynamic therapy (aPDT; also called antimicrobial chemotherapy, PACT) (see Table 1) [26]. In a photodynamic reaction, a light-sensitive molecule (photosensitizer) is supplied, which becomes reactive in the presence of light. A prominent group of photosensitizers are porphyrins and among them, chlorophyll is probably the one with the highest abundance, the easiest to obtain and therefore the cheapest. In particular, for metalloporphyrins it has been shown that they are accumulated very effectively by bacteria via heme uptake systems [27]. Photoactivation of photosensitizers leads to formation of reactive oxygen species (ROS). Upon light excitation an electron of the photosensitizer molecule is transferred from the ground state to an activated singlet state. This state has a short lifetime and the excited electron returns to the ground state again via internal conversion releasing the excitation energy as fluorescence photon or heat, respectively. The other possibility is the conversion via intersystem-crossing to the less energetic but long-living triplet state. From there the electrons return to the ground state by emitting a phosphorescence photon (longer wavelength compared to fluorescence) or may interact with oxygen in two different ways: (1) In a Type I photochemical reaction, the transfer of the activated electron onto an oxygen molecule leads to the formation of superoxide radicals, which forms a variety of other ROS, such as hydrogen peroxide (H_2_O_2_) or highly reactive hydroxyl radicals (^•^OH). (2) In a Type II photochemical reaction, an exclusive transfer of energy onto molecular oxygen, leads to formation of high-reactive singlet oxygen (^1^O_2_) [28,29]. Both mechanisms can occur simultaneously but, in most cases, aPDT proceeds via a Type II reaction. Introduced to a cell, damages on various levels can occur, possibly resulting in cell death [30]. Bactericidal antibiotics were also associated with ROS as a prolonged production of ROS during bacterial killing was assumed [31], but ROS alone cannot explain the antibiotic activity [32,33].

In general, Gram-negative bacteria are less susceptible to aPDT compared to Gram-positive species. While Gram-positives are surrounded by a thick but porous peptidoglycan layer, in Gram-negatives the complex and impermeable outer membrane with lipopolysaccharides limits the entrance of anionic or neutral-charged molecules [34]. Cationic photosensitizers may penetrate this permeability barrier, but low water solubility and aggregation often counteract phototoxicity by reduced singlet oxygen quantum yields [35]. Type I reactions are favored when targeting Gram-negative bacteria because these cells were found to be more susceptible to ^•^OH than to ^1^O_2_ [36].

Due to a self-produced polymeric matrix, bacteria biofilms are much more resistant to antimicrobial agents compared to planktonic cultures [37]. Experiments with monospecies biofilms by *Enterococcus faecalis* and *Actinomyces naeslundii* as well as with polymicrobial biofilms clearly indicated that phenalenone-based aPDT can be a promising method to control bacteria biofilms (Table 1) [38,39]. The photosensitizer SAPYR (2-((4-pyridinyl)methyl)-1H-phenalen-1-one chloride) destroyed bacteria, embedded in extracellular polymeric substance to an extent that is regarded as disinfection [38]. In addition, methylene blue in combination with a low-intensity laser was successfully used to control cariogenic-like biofilms formed by *Streptococcus mutans* [40].

Initially, our group investigated the possible use of chlorophyllin, a hydrophilic chlorophyll derivative with a singlet oxygen quantum yield of Φ_Δ_ = 0.3 [54], against fish parasites, snails, and mosquito larvae in vitro and in situ [55,56]. The results were promising and designated chlorophyllin for application in aquaculture as well as against waterborne vectors for human parasites such as snails (e.g., schistosomiasis) or mosquitoes (e.g., malaria or various arboviroses).

In the current study, we dealt with the major thread of spreading resistances in pathogenic bacteria. Therefore, we wanted to take a closer look into the possible use of chlorophyllin-based photosensitization to control the growth of Gram-positive and Gram-negative bacteria. For our investigations we used model strains: *Bacillus subtilis* 168, *Escherichia coli* DH5α, and the *E. coli* mutant strain NR698 with a deficient outer membrane mimicking a “Gram-positive cell envelope”.

## 2. Materials and Methods

### 2.1. Bacteria Strains and Cell Culture

Experiments were performed with *E. coli* DH5α (Invitrogen, Carlsbad, CA, USA), *E. coli* NR698 (MC4100 *lptD4213*; kindly provided by M. Grabowicz, Princeton University, NJ, USA) [57] and *B. subtilis* 168 (*trpC2*; laboratory stock). Bacteria cells were grown in standard lysogeny broth (LB) medium [58] overnight in an incubator (37 °C, 150 rpm). Prior to the experiments, cell concentration was determined optically at 590 nm and set to OD_590_ = 0.1 before cells were diluted in LB as required. During the experiments, cells were grown on/in LB at 37 °C if not indicated otherwise. Cells were placed on a bright surface and temperature was constantly controlled inside a climate cabinet to avoid sample heating upon illumination. Prior to the experiment, the temperature within the cabinet was measured simultaneously at 5 points and the airflow modified until homogenous temperature was achieved.

### 2.2. Illumination

**Figure 1:**
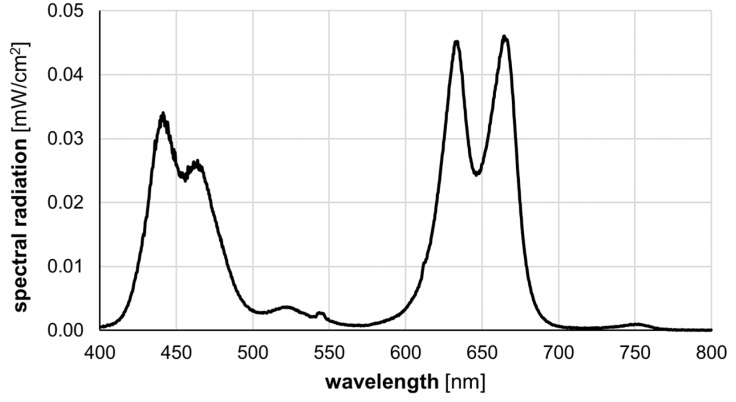
Light emission spectrum for PRAKASA 300 W.

Bacteria were irradiated with the LED grow light PRAKASA 300 W (Green Tech Direct Ltd., Harrow, Middlesex, UK). Distance to samples was adjusted to achieve a photon flux of 560 µE/(s × m²) which corresponds to a light intensity of 12 mW/cm^2^. The spectral radiation of the light source in shown in Figure 1. Lower light intensity was regulated with neutral density filters (Lee-Filters, Andover, Hampshire, UK).

### 2.3. Chlorophyllin Extraction

**Figure 2:**
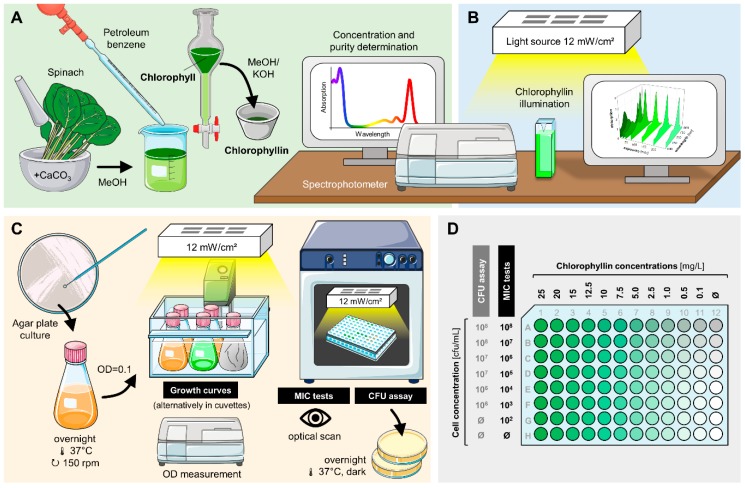
Overview of experimental procedures. (**A**) Chlorophyllin extraction from spinach. (**B**) Determination of chlorophyllin stability in light. (**C**) Experimental setup to test the effects of chlorophyllin on the growth and viability of different bacteria. (**D**) 96-well matrix plate layout for Colony-forming units (CFU) assays and the determination of minimum inhibitory chlorophyllin concentrations (MIC test). The final volume of each well was 200 µL. After inoculation, the plate was incubated at 37 °C in large plastic bags, saturated with water vapor. Parts of the figure were drawn by using pictures from Servier Medical Art, licensed under a Creative Commons Attribution 3.0 Unported License (https://creativecommons.org/licenses/by/3.0/).

Chlorophyll from frozen spinach leaves was extracted at room temperature in darkness with 100% methanol (MeOH; VWR International, Radnor, PA, USA) (Figure 2A). To 1 kg plant material 5 g CaCO_3_ (Carl Roth, Karlsruhe, Germany) was added to avoid acidification. The filtrate was mixed with petroleum benzene (boiling range 40–60 °C; Carl Roth). Chlorophyll became enriched in the upper lipophilic phase, which was separated using a separation funnel. 5 mL of 100 mM methanolic KOH (AppliChem, Darmstadt, Germany) were added to convert chlorophyll into water-soluble chlorophyllin, which moved from the benzene phase into the MeOH/KOH phase. Concentration of chlorophyllin was determined spectrophotometrically using the empiric formulas of Lichtenthaler and Wellburn [59]. Spectra did not indicate other substances absorbing in UV or visible wavelength range (data not shown). Aliquots were stored in darkness at −20 °C prior to use. To avoid transfer of MeOH to the samples, volumes which contained the amount of chlorophyllin needed for experiments were evaporated in darkness. Remaining chlorophyllin powder was dissolved in LB medium to a working solution of 50 mg/L without the pH changing. The successive steps of this isolation procedure (different phases and chlorophyll-modification in the final step) make the method very suitable to obtain pure chlorophyllin.

To verify that the observed effects are chlorophyllin-dependent some additional experiments were conducted with commercially available chlorophyll (Sigma-Aldrich, Steinheim, Germany), which was converted into chlorophyllin as described.

### 2.4. Chlorophyllin Stability in Light

Chlorophyllin (25 mg/L) in LB medium was illuminated with 12 mW/cm^2^. Samples were drawn at several time points (0 min, 15 min, 30 min, 1 h, 2 h, 3 h, 4 h). Absorption spectra were recorded with a UV-2550 spectrophotometer (Shimadzu, Kyōto, Japan). In addition, chlorophyllin concentrations were determined as previously described by Wohllebe et al. [56] (Figure 2B).

### 2.5. Growth Experiments

Preliminary experiments were performed with 50 mL LB cell suspension in 100 mL Erlenmeyer flasks inside a waterbath shaker at 37 °C (n = 2) (Figure 2C). Samples for dark incubation were covered with aluminum foil. Initial cell concentration was adjusted to 10^6^ cells/mL. Initial chlorophyllin concentration was kept at 22 mg/L (0.04 mM). Light intensity for illuminated samples was 12 mW/cm^2^. Samples were drawn in defined time intervals and measured photometrically at 590 nm.

For further growth experiments cells were incubated in 1 mL polystyrene cuvettes (Sarstedt, Nümbrecht, Germany). Different chlorophyllin concentrations (0.01–25 mg/L; 0.1–100 mg/L for *E. coli* DH5α) were tested (n = 3). Cell-free LB medium with corresponding chlorophyllin concentrations served as blanks. Cuvettes were put in a sterile plastic bag to avoid contamination. They were illuminated (12 mW/cm^2^) or protected from light, incubated on a shaker at 37 °C. Cell growth was determined photometrically at 590 nm (OD_590_) prior to exposure (0 min), and subsequently after 30 min, 60 min, 120 min, 180 min, and 24 h.

### 2.6. Determination of Effective Chlorophyllin Concentrations

Determination of minimum inhibitory concentrations (MICs) were performed in 96-well plates (Sarstedt). Cells, chlorophyllin solution (50 mg/L) and LB medium were mixed in different proportions into the wells of the matrix plate (n = 3) (see Figure 2D). Dark control plates were covered with aluminum foil. To avoid evaporation all plates were inserted in plastic bags together with a humid towel, leaving a sufficient air reservoir. Bags were placed on a shaker (150 rpm) inside an incubator (37 °C, or 28 °C/42 °C for temperature experiments) and illuminated at 12 mW/cm^2^. After 24 h (total light dose: ~1000 J/cm^2^) plates were analyzed optically for bacteria growth.

Using a cell number of 10^6^ cfu/mL, the necessary exposure to light for different chlorophyllin concentrations was determined. *E. coli* DH5α cells in LB medium with different concentrations of chlorophyllin were filled into petri dishes (Sarstedt) and exposed to light (12 mW/cm^2^). Samples were drawn in regular time intervals and mixed with fresh LB medium, then incubated overnight at 37 °C. The test point at which no cell growth was detected (clear LB medium after incubation) was regarded as the minimal time of exposure for complete inactivation.

To determine the effects of different light intensities, *E. coli* DH5α (cell number: 10^6^ cfu/mL) was incubated in LB with 25 mg/L (0.04 mM) chlorophyllin and filled in petri dishes. To reduce the light intensities some of the samples were covered with neutral density filters (Lee-Filters; transmission: 70%, 50%, 25% and 12.5%, respectively). In regular time intervals, samples were drawn, mixed with fresh LB medium and incubated overnight at 37 °C as described above.

### 2.7. Colony-Forming Units (CFU) Assay

Samples were prepared in 96-well plates (Sarstedt) (each 200 µL cell suspension per well). Following cell concentrations were prepared for all bacteria strains: 10^8^, 10^7^ and 10^6^ cfu/mL. Concentrations of chlorophyllin was between 0–25 mg/L (plate preparation see Figure 1D). Two identical 96-well plates were pipetted, one served as light experiment the other one (covered with aluminum foil) served as dark exposure experiment. The plates were incubated at 37 °C under light (12 mW/cm^2^). Prior to exposure (0 min), and to defined time points (30 min, 60 min, 90 min, 120 min, 180 min, 24 h) volumes of 2.5 µL from each well were withdrawn and transferred on rectangular LB agar plates. After 24 h of incubation at 37 °C (darkness) the plates were scanned for colonies.

### 2.8. Fluorescence Microscopy

To visualize chlorophyllin uptake in *E. coli*, cells were cultured in LB medium in presence of 25 mg/L chlorophyllin for 20 min. Cells were harvested by centrifugation and washed with fresh LB medium twice before they were visualized using the Biozero BZ-8000 closed digital and inverted fluorescence microscope (Keyence, Osaka, Japan). Fluorescence images were taken with a 100× oil immersion objective, an excitation wavelength of 450–490 nm and an emission cut-off at 510 nm.

### 2.9. Data Evaluation and Statistics

The optical densities of corresponding blanks were subtracted from the optical densities of the samples. Subsequently mean values as well as standard deviation of the triplicates were calculated and presented graphically. To analyze differences between samples and controls, corresponding means of controls were subtracted by samples means. In addition, differences between samples and corresponding controls were determined with an independent-samples t-test using the IBM SPSS Statistics 23.0 software (IBM Deutschland GmbH, Ehningen, Germany). *p* < 0.05 was considered as statistically significant.

Cell numbers were calculated assuming that OD_590_ of 0.1 corresponds to 0.8 × 10^8^ cfu/mL. Growth rate from one time point (*t*_1_) to the next (*t*_2_) was determined using Equation (1).

(1) $$divisions\;t_{2}-t_{1}=\frac{\log\left(t_{2}\right)-\log\left(t_{1}\right)}{\log\left(2\right)},$$

divisions, where divisions *t*_2_ − *t*_1_ = number of duplications, *t*_2_ = new time point, *t*_1_ = previous time point.

Determination of EC_50_-values: The means of all related *LB* controls were calculated. The inhibition-values in all particular sample values and the single *LB* controls of the corresponding experiment compared to the LB-means were determined according to following formula:

(2) $$Inhib\left(\%\right)=-\left(\left(\frac{sample\;value}{LB-mean}\times 100\right)-100\right)$$

All corresponding inhibition-values were used to calculate dose-effect relationships and EC50 values by using a sigmoid equation with three parameters:

(3) $$y=\frac{a}{1+e^{-\left(\frac{x-x_{0}}{b}\right)}},$$

where *y* is the response variable (inhibition of a given parameter), *a* is the difference between maximum and minimum values in the curve, *b* is the slope of the curve, *x* is the corresponding chlorophyllin concentration, *x*_0_ is a vertical shift factor. Data were processed using the software SigmaPlot 8.0 for Windows 2000 (IBM, Armonk, NY, USA). SigmaPlot delivered the best approximation for *a*, *b*, and *x*_0_. To determine the EC_50_-values, respectively the formula was solved for *x*. With *y* = 50% *x* is the EC_50_-concentration:

(4) $$x=-\left(\frac{a}{y}-1\right)\times b+x_{0}$$

## 3. Results

### 3.1. A Question of Gram: Effects of Chlorophyllin on the Early Growth Behavior of Different Bacteria

In a first approach to determine the effects of purified chlorophyllin on different bacteria species, growth of the Gram-negative model strain *E. coli* DH5α and the Gram-positive model strain *B. subtilis* 168 were investigated under different conditions starting with a cell concentration of 10^6^ cfu/mL:

- illuminated cells in LB
- non-illuminated cells in LB
- illuminated cells in LB with evaporated solvent (MeOH/KOH)
- illuminated cells in the presence of 22 mg/L chlorophyllin
- non-illuminated cells in the presence of 22 mg/L chlorophyllin

**Figure 3:**
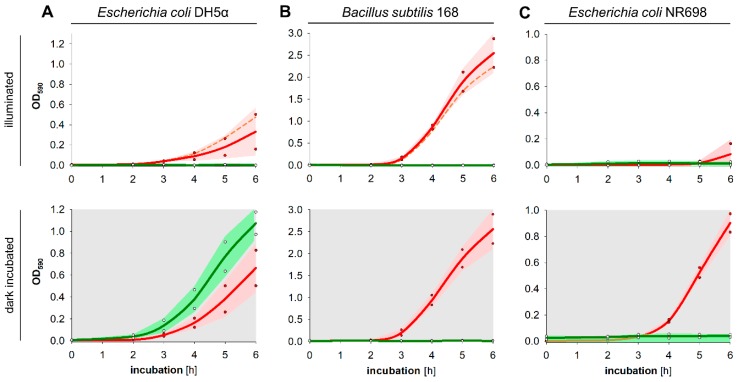
Effect of chlorophyllin on the early growth phase of (**A**) *Escherichia coli* DH5α, (**B**) *Bacillus subtilis* 168, and (**C**) *Escherichia coli* NR698. Liquid cultures of bacteria (initial cell number: 10^6^ cfu/mL) were incubated in Erlenmeyer flasks in standard LB medium (red) and in presence of a chlorophyllin concentration of 22 mg/L (green). Cells grew either illuminated with 12 mW/cm^2^ (bright plots, upper row) or in darkness (grey plots, lower row). Depicted are measured values (circles) and fitted curves (solid lines) with corresponding 95% confidence limits (red and green areas). Dashed lines describe growth of example cultures in LB medium + MeOH/KOH (solvent of chlorophyllin).

Optical density of the samples was measured in regular time intervals up to 6 h. Growth of *E. coli* and *B. subtilis* was slightly affected by illumination compared to the dark exposed cultures, although this effect was smaller for *B. subtilis* than for *E. coli* (Figure 3A,B). In presence of chlorophyllin and light, proliferation of Gram-negative *E. coli* was completely inhibited, whereas chlorophyllin in darkness seemed to support growth (Figure 3A). Gram-positive *B. subtilis* in contrast was completely inactivated by chlorophyllin both in light and in darkness (Figure 3B).

As we supposed that the outer membrane of Gram-negative bacteria may be a barrier for chlorophyllin uptake, we additionally tested *E. coli* NR698, which has a defective outer membrane. This strain also showed a much slower proliferation in light. Similar to *B. subtilis*, growth of *E. coli* NR698 was inhibited in presence of chlorophyllin both in illuminated cultures and in cultures protected from light (Figure 3C).

### 3.2. Determination of the Minimal Inhibitory Chlorophyllin Concentration

After the promising results of the preliminary growth experiments, the sensitivity of the three bacterial strains (*E. coli* DH5α, *B. subtilis* 168 and *E. coli* NR698) against different chlorophyllin concentration was determined in light (total light dose: ~1000 J/cm^2^) and darkness. At high cell densities (10^7^-10^8^ cfu/mL), growth of the *E. coli* “wildtype” strain DH5α was not completely inhibited even at the highest concentration used (25 mg/L), while growth of *B. subtilis* and *E. coli* NR698 was inhibited both in light and in darkness (Table 2). To exclude effects of the extract not related to chlorophyllin, experiments with chlorophyllin made of commercial purified chlorophyll were performed, which showed very similar results although the effect was slightly stronger (Table 3). To clarify if the photodynamic effect of chlorophyllin in killing bacteria does require an aerobic milieu, matrix plates with *E. coli* were cultured both under aerobic and anaerobic conditions. Here we could detect no difference between the MICs of aerobic and anaerobic culturing (data not shown).

**Figure 4:**
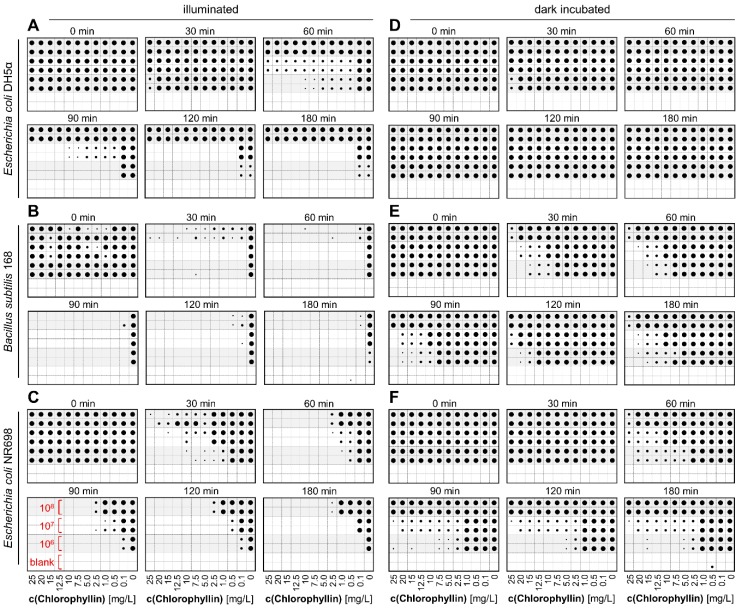
Schematic presentation of evaluation of CFU ability after incubation to chlorophyllin. Differently dense liquid cultures of (**A**) *Escherichia coli* DH5α, (**B**) *Bacillus subtilis* 168, and (**C**) *Escherichia coli* NR698 were supplemented with different chlorophyllin concentrations between 0.1 and 25 mg/L. Cells grew in 96-well matrix plates either illuminated with 12 mW/cm^2^ (**A**–**C**) or protected from light (**D**–**F**). Samples (2.5 µL) were drawn at different time points and transferred onto LB agar plates. After overnight incubation at 37 °C in the dark, colony growth was analyzed. Dot size quantifies colony growth. Original pictures of the agar plates can be found in Figure S1.

To investigate inactivation time, CFU ability of the three bacterial strains at different cell numbers and chlorophyllin concentrations was tested in subsequent time intervals. At each time point aliquots of the cell suspensions were transferred to LB agar plates. After overnight incubation, growth of colonies was analyzed. In case colony growth was visibly impaired (smaller colony size compared to control) cells were regarded as affected. The lowest concentration at which no more colonies were found (or colonies were impaired) are visualized in Figure 4. At 10^8^ cfu/mL *E. coli* DH5α was not affected by chlorophyllin neither in light (Figure 4A) nor during incubation in darkness (Figure 4D). At lower cell numbers (10^6^–10^7^ cfu/mL) increasing incubation time in light resulted in decreasing CFU ability of cells, beginning with high chlorophyllin concentrations to low concentrations. After 180 min of exposure only at 0.1 mg/L chlorophyllin colonies were found. Less dense cultures (10^6^ cfu/mL) were already affected by light without presence of chlorophyllin. In darkness, no detrimental growth effect was observed.

Already after short time (60 min) *B. subtilis* samples were affected at all chlorophyllin concentrations when illuminated (Figure 4B). In darkness, *B. subtilis* was less sensitive against chlorophyllin. At a cell number of 10^8^ cfu/mL colony formation was inhibited at 15–20 mg/L chlorophyllin. Less dense cultures (10^6^ cfu/mL and 10^7^ cfu/mL) formed colonies at all concentrations ≤25 mg/L (Figure 4E).

*E. coli* NR698 is more sensitive against chlorophyllin compared to *E. coli* DH5α. In light, colony formation became stronger inhibited over time. After 180 min also at the lowest concentration no colony growth was detected in 10^6^ cfu/mL samples. 10^7^ cfu/mL samples showed colonies below 0.5 mg/L and at cell number of 10^8^ cfu/mL threshold was below 5 mg/L chlorophyllin (Figure 4C). Protected from light, cell suspensions with 10^7^ cfu/mL and 10^6^ cfu/mL were inhibited at 25 mg/L or 7.5 mg/L, 10^8^ cfu/mL samples formed colonies at all chlorophyllin concentrations (Figure 4F).

### 3.3. Examining the Limits of a Photodynamic Therapy With Chlorophyllin

#### 3.3.1. Chlorophyllin Concentration

To test the concentration-dependent effect of chlorophyllin on the growth of *E. coli* DH5α, *E. coli* NR698 and *B. subtilis* 168, cell suspension cultures were adjusted to an OD_590_ of 0.1 (initial cell number of about 10^8^ cfu/mL) and incubated in presence of different chlorophyllin concentrations between 0.1 and 25 mg/L.

**Figure 5:**
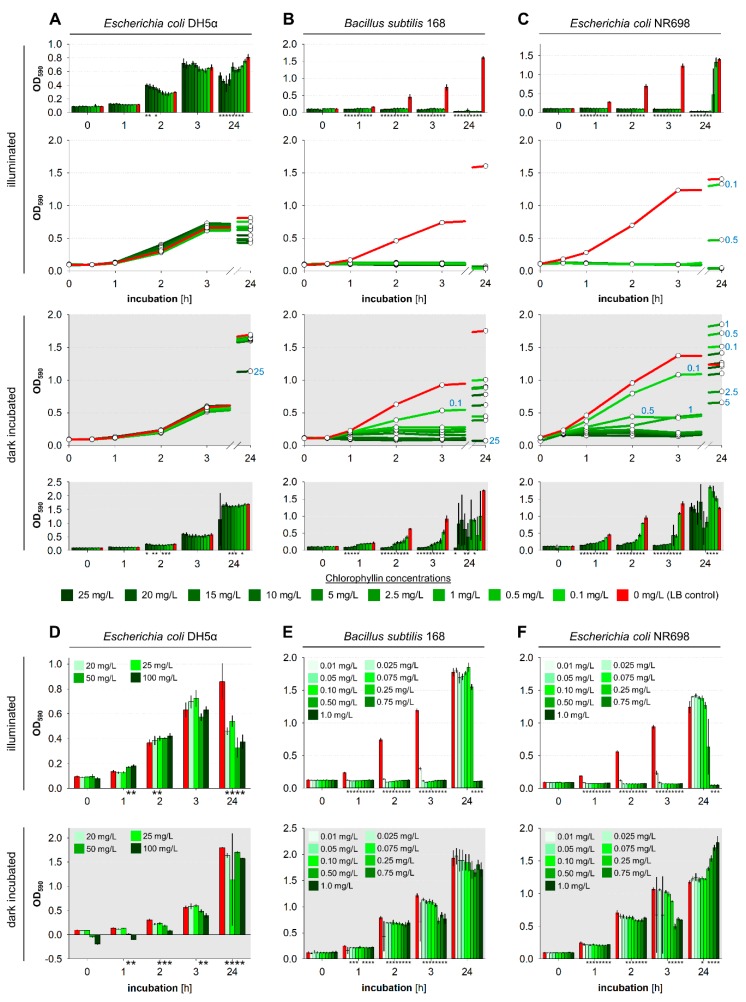
Influence of different chlorophyllin concentrations on the growth of (**A**) *Escherichia coli* DH5α, (**B**) *Bacillus subtilis* 168 and (**C**) *Escherichia coli* NR698 (initial cell number: ~10^8^ cfu/mL). Bacteria were cultured for 24 h at 37 °C exposed to light (12 mW/cm^2^; bright plots, upper row) or in darkness (grey plots, lower row). To determine the lower limit of efficacy, additional chlorophyllin concentrations were tested: (**D**) *Escherichia coli* DH5α, 20↑100 mg/L (**E**) *Bacillus subtilis* 168, 1.0↓0.01 mg/L and (**F**) *Escherichia coli* NR698, 1.0↓0.01 mg/L. Depicted are means (growth curves) together with corresponding 95% confidence limits (bar charts). Blue numbers indicate respective chlorophyllin concentrations. * *p* < 0.05 vs. control culture without chlorophyllin.

For *E. coli* DH5α no inhibition of cell growth was found within 3 h of incubation treated with ≥25 mg/L chlorophyllin (Figure 5A). In contrast, in the presence of higher chlorophyllin concentrations an increased growth was observed. After 24 h of incubation in light samples treated with ≥0.5 mg/L chlorophyllin showed a reduced cell number compared to the controls. In darkness, a slight reduction in cell number was observed only at the highest concentration tested (25 mg/L) (Figure 5A). Even at excessive chlorophyllin concentrations (50–100 mg/L) no significant differences between chlorophyllin samples and controls were detected in the first 3 h (Figure 5D). Consistent with the lower concentrations the illuminated chlorophyllin samples showed a reduced cell number after 24 h (Figure 5D).

Growth of *B. subtilis* was almost completely suppressed in illuminated cultures at all chlorophyllin concentrations down to 0.5 mg/L (Figure 5B). At very low concentrations in the range of 0.01 and 0.25 mg/L we found a delayed cell growth between 3 and 24 h (Figure 5E). In darkness, growth inhibition was found starting from a chlorophyllin concentration of 5 mg/L. EC_50_-values were calculated in ranges of 0.01–0.001 mg/L for light-exposed samples (depending on exposure time) and 0.8–2.7 mg/L for samples protected from light (Table 4).

Effect of chlorophyllin on *B. subtilis* and *E. coli* NR698 was almost identical. Compared to DH5α, *E. coli* NR698 was much more sensitive against chlorophyllin both in light and in darkness. In light, bacterial growth was almost completely inhibited at all tested concentrations down to 0.5 mg/L (Figure 5C,F). In darkness, growth inhibition became obvious already at chlorophyllin concentrations of 0.5-1 mg/L within the first 3 h before the inhibitory effect was reduced after 24 h, similar to observations made with *B. subtilis* (Figure 5C). At this time growth is even enhanced by chlorophyllin concentrations between 0.25 and 1 mg/L (Figure 5F). The calculated EC_50_-values for the different time points are shown in Table 4.

#### 3.3.2. Chlorophyll Degradation

**Figure 6:**
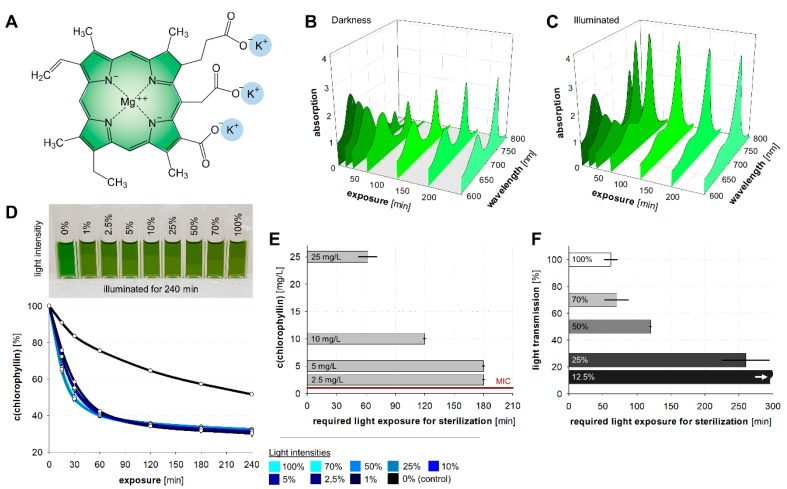
(**A**) Chemical structure of isolated chlorophyllin. (**B**) Alterations of chlorophyllin’s absorption spectrum in darkness and (**C**) exposed to light with 12 mW/cm^2^. (**D**) Reduction of chlorophyllin concentration exposed to different light intensities. (**E**) Required light exposure times to sterilize an *E. coli* DH5α suspension culture initial cell number: ~10^6^ cfu/mL) with a light intensity of 12 mW/cm^2^ using different chlorophyllin concentrations. (**F**) Required light exposure times to sterilize an *E. coli* DH5α suspension culture (initial cell number: ~10^6^ cfu/mL) with a chlorophyllin concentration of 25 mg/L and different light intensities. For 12.5% light intensity no sterilization was observed within 300 min. Depicted are means ± standard deviation.

In vivo, chlorophyll molecules are embedded in a defined complex of proteins, which provide a supply of electrons as substitute for the ones passed on to the electron transport chain, and other pigments, which absorb surplus energy. In vitro, these vectoral electron transport and safety functions are missing, and chlorophyll degrades upon illumination in the presence of oxygen [60]. This is indicated by a gradual loss of its green color, as the molecules break down to smaller colorless moieties. The most prominent among them are glycerol, carbonic acids (lactic, succinic, oxalic, malonic, and citric acid), alanine, and methyl ethyl maleimide [61]. To evaluate the lifetime of the chlorophyllin preparation (Figure 6A), we irradiated 25 mg/L in LB medium with different intensities for four hours and measured the concentration of intact chlorophyllin as a function of the absorption. The alterations of chlorophyllin’s absorption spectrum in LB medium exposed to/protected from light are shown in Figure 6B,C. A quick decrease in chlorophyllin concentration occurred in the first 60 min of illumination regardless of its treatment before the decline slowed down (Figure 6D). The dark control decreased also markedly, which is probably caused by oxidation.

#### 3.3.3. Light Exposure Time

In a next step we determined the necessary light exposure time for sterilization using different chlorophyllin concentrations. With an initial chlorophyllin concentration of 25 mg/L an exposure time to light of about 1 h (42 J/cm^2^) was necessary for complete bacteria inactivation. At lower concentrations, the necessary exposure time increased to 2 h (10 mg/L) and 3 h (7.5 or 5 mg/L) (Figure 6E).

#### 3.3.4. Light Intensity

At high light intensities of 12 mW/cm^2^ (no filter) and 8.4 mW/cm^2^ (T = 70%) the necessary exposure time for complete inactivation (no cell growth after overnight incubation) was about 1 h (Figure 6F). At a lower light intensity of 6 mW/cm^2^ (T = 50%) about 2 h were necessary and at 3 mW/cm^2^ (T = 25%) about 4 h of exposure were necessary to inactivate the cells. At 1.5 mW/cm^2^, no inactivation was observed within 5 h (Figure 6F).

#### 3.3.5. Temperature

To investigate a possible temperature-dependent efficacy of chlorophyllin, we analyzed bacterial growth of the most sensitive strain *B. subtilis* 168 additionally at 28 °C and 42 °C. Cells were incubated in LB with different concentrations of chlorophyllin and exposed to light (12 mW/cm^2^). We observed a tended decrease of chlorophyllin MICs at 28 °C and an increase at 42 °C in darkness (Table 5).

## 4. Discussion

### 4.1. Photodynamic Treatment with Porphyrins

Experiments dealing with aPDT of bacteria yield very promising results. In particular, experiments with porphyrins against pathogens such as methicillin-resistant *Staphylococcus aureus* showed that porphyrins are very effective in bacteria inactivation [48]. The antimicrobial activity is based on the chemical properties of porphyrins, e.g., the ability to transfer electrons, catalyze peroxidase, and oxidase reactions, absorb photons, generate ROS and the partition into lipids of bacterial membranes [27]. In a recent experiment, Buchovec et al. [62] could clearly demonstrate that the photodynamic effect of chlorophyllin on *Salmonella enterica* is mainly caused by oxidative stress, because addition of NaN_3_ (a known quencher of singlet oxygen) drastically increases the survival rate. In the same study, transcription of 33 stress-related genes was investigated by quantitative RT-PCR, indicating expression changes or genes involved in oxidative stress [62].

In 2008, Maclean et al. [63] reported that the photoinactivation of *S. aureus* is highly dependent on the oxygen concentration. They postulated that at higher bacterial concentrations oxygen may be consumed faster than it is resupplied by diffusion through the sample surface and this would lead to a lower bacterial photoinactivation. This we could not detect and consistent with our result, dissolved oxygen seemed not to affect the success of photoinactivation of *Vibrio fischeri* using a tricationic *meso*-substituted porphyrin in aquaculture [64].

### 4.2. The Outer Membrane Is a Barrier That Often Impedes Photodynamic Therapies

The outer membrane of Gram-negative bacteria is a barrier for many hydrophobic and larger hydrophilic substances (>600 Da) [65] what especially impacts the activity of several antibiotics: Macrolides, novobiocin, rifamycin, lincomycin, clindamycin, or fusidic acid are highly effective against Gram-positives, but show only negligible efficacy against Gram-negative bacteria [66]. Consequently, infections with Gram-negative bacteria have been much more prevalent, even in modern hospitals for quite some time [67]. Beyond that, the efficacy of porphyrin-based aPDT is influenced by the outer membrane. Early approaches with hematoporphyrin demonstrated that a concentration of 0.1–1 mg/L can inactivate more that 99.9% of *Streptococcus faecalis* bacteria in culture, when they are incubated for 10 min in white light [68,69]. Similar results were obtained with other Gram-positives such as *S. aureus*, *Streptococcus pyogenes*, *Bacillus cereus*, *Propionibacterium acnes*, *Enterococcus hirae*, as well as with mycoplasmas and yeast cells [70,71,72]. In contrast, Gram-negative bacteria seemed to be resistant to these treatments [73,74]. Banfi et al. [75], who synthesized and tested various tetraaryl-porphyrin molecules on different bacteria strains, also found higher sensitivity of Gram-positive bacteria against porphyrin-based substances: the Gram-positive *S. aureus* was much more sensitive compared to the Gram-negative *E. coli* and *P. aeruginosa* strains. Our experiments confirmed that *E. coli* is far less sensitive against chlorophyllin treatment compared to the Gram-positive *B. subtilis*. The reason for the lower sensitivity of Gram-negative bacteria is most likely the outer membrane, as *E. coli* NR698 showed a comparable sensitivity against chlorophyllin as *B. subtilis* indicating that this membrane prevents accumulation of chlorophyllin in the cytoplasmic membrane. First hints for the imputation of the outer membrane were gathered by Nitzan et al. [76], who combined polymyxin nonapeptide as a membrane-destabilizing agent with deuteroporphyrin, demonstrating a light-dependent activity of a porphyrin against *E. coli* and *P. aeruginosa* for the first time.

To avoid this permeability problem, today, porphyrin-based photosensitization of Gram-negatives is often combined it with other antimicrobial or membrane-destabilizing treatments. First success was reported for a chlorophyllin-chitosan complex in the treatment of *S. enterica* [62,77]. In addition to chitosan, positively charged substances such as ethylenediaminetetraacetic acid (EDTA), large cationic molecules, or polymers such as compound 48/80 can disorganize the outer membrane of Gram-negative bacteria [78] counteracting the negatively charged cell wall and lipopolysaccharides (LPS). This way, they can act as door openers for photosensitizers. Polyethyleneimine (PEI), an aliphatic polycationic polymer increases membrane permeability but did not affect viability of the bacteria [79]. Dei et al. [80] used a PEI-chlorin e6 conjugate to treat MRSA-infected skin abrasion wounds of mice successfully.

Wainwright et al. [81] have synthesized derivatives of the photosensitizer methylene blue. Some of these exhibited a strong effect in light as well as in darkness, while others such as 3-(bis(3-(dimethyl-amino)-propyl)amino)-7-(di-n-propylamino) phenothiazinium iodide showed activity only in light. Using water-soluble zinc pyridinium phthalocyanine (PPC) as a photodynamic agent, both *E. coli* and *P. aeruginosa* could be inactivated in light. The presence of 10 mg/L PPC resulted in a 5 log_10_ decrease of cell survival in *E. coli* after 60 min illumination (1 mW/cm^2^ in the range 600-700 nm). In *P. aeruginosa*, 25 mg/L PPC were necessary to induce the same reduction under identical light conditions.

Toluidine blue O and porphyrin were successfully tested in bacteria (*Prevotella melaninogenica*, *Porphyromonas gingivalis*, and *Aggregatibacter actinomycetemcomitans*) responsible for periodontitis, an infection of the gums [43]. By means of portable LED illumination, survival rate of the three Gram-negative strains could be reduced significantly, although no complete disinfection was achieved. This was a very promising result as Gram-negatives are most likely less sensitive to photodynamic treatment.

The porphyrin TDPyP (5-[4-(1-dodecanoylpyridinium)]-10,15,20-triphenyl-porphyrinyl chloride) did not show drastic effects on bacteria survival if applied directly. In contrast, incorporation in a polycationic liposome with DOTAP (N-[1-(2,3-Dioleoyloxy)propyl]-N,N,N-trimethylammonium methyl-sulfate) increased deactivation enormously. Already after 5 min incubation with 5 µM of DOTAP-bound TDPyP, 10 min irradiation with 50-100 mW/cm^2^ resulted in dramatic decrease in cell survival [48]. The study by Banfi et al. [75] found that three of the tested tetraaryl-porphyrins showed considerably strong effects on survival rate of *E. coli*, *S. aureus* and *P. aeruginosa*. 5,10,15,20-Tetra(N-benzyl-4-pyridyl)-porphyrin tetrachloride was the most effective compound.

### 4.3. Chlorophyllin Is Not Only a Photosensitizer: The Two Modes of Action

**Figure 7:**
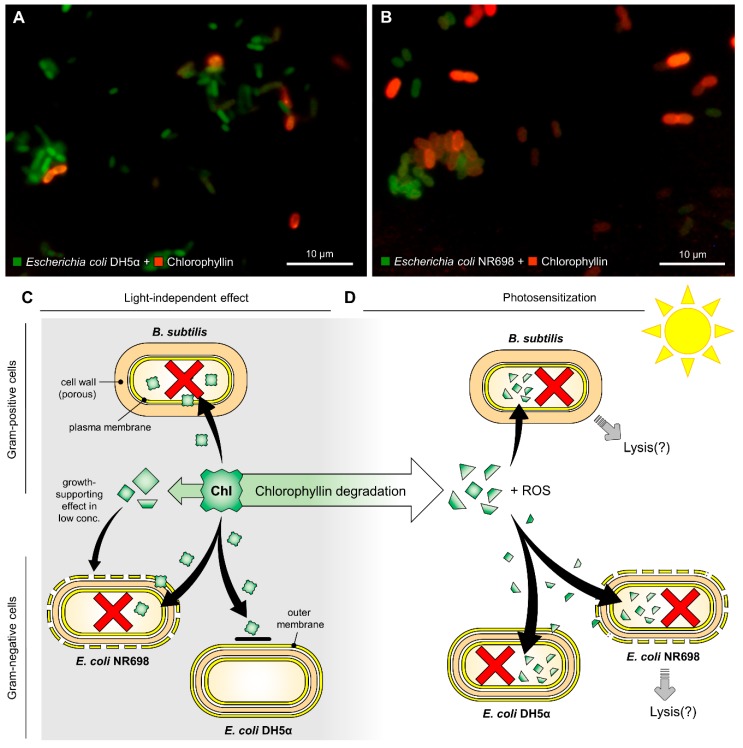
Fluorescence microscopic images of (**A**) *Escherichia coli* DH5α and (**B**) *Escherichia coli* NR698 after exposure to chlorophyllin (red fluorescence). Assumed modes of action of chlorophyllin against Gram-positive and Gram-negative bacteria. (**C**) Chlorophyllin (Chl) molecules cannot pass the intact outer membrane of Gram-negative bacteria. (**D**) Chlorophyllin is degraded in light. Degradation products can enter both Gram-negative and Gram-positive cells. The red crosses indicate cell death.

The discovery that chlorophyll acts as a photosensitizer and can kill bacteria is not completely new [62,82]. Nevertheless, our data clearly confirmed that even the water-soluble derivative chlorophyllin inactivates both Gram-positive and Gram-negative bacteria strains upon irradiation. Gram-positives such as *B. subtilis* are more susceptible to this inactivation. A completely new finding was that beyond photosensitization *B. subtilis* also showed a considerable inactivation in darkness. This was supported by results obtained with *E. coli* NR698 comprising a deficient outer membrane, indicating that the “dark-active” component of chlorophyllin cannot pass the outer membrane of Gram-negatives (Figure 7).

The lower chlorophyllin sensitivity found for *B. subtilis* cultured at 42 °C seems to be consistent with current observations that an increase in temperature of 10 °C can be associated with an increase in antibiotic resistance of common pathogens [83]. This effect could be explained on the one hand by temperature-dependent modifications of bacterial metabolism (regardless of the mechanism of action of the antibiotic agent used) or on the other hand by alterations in proteins and/or LPS. Similar effects were observed with the uptake of antibiotics in *Stenotrophomonas* (*Xanthomonas*) *maltophilia* [84]. For this species, a correlation between the LPS pattern and a decreased susceptibility to aminoglycosides was found that strongly suggested that the growth temperature affects the LPS composition challenging the binding or the uptake of macromolecules. The photodynamic effect of chlorophyllin on bacteria seems to be more complex as the quick decrease in chlorophyllin concentration did not reflect in the growth curves. In addition, the growth-promoting effect of chlorophyllin in concentrations between 0.25 and 1.0 mg/L that was found in 24 h experiments with *E. coli* NR698 requires further analyses. The degradation of chlorophyllin to different carbonic acids, glycerol and alanine [61] which can be used as nutrients in a sub-toxic environment could possibly explain the positive effect on cell growth. A further interesting observation was the OD-reduction of *E. coli* NR698 and *B. subtilis* cultures treated with chlorophyllin in light (see Figure 5B,C). This indicates not only a bactericidal effect but also the lysis of cells. There is still some debate about whether extracellular or intracellular ROS is more effective in killing bacteria by aPDT. ROS formation may affect cells on various levels such as membranes, proteins and DNA [66]. However, it has been described that uptake of a photosensitizer is not always necessary to inactivate bacteria [39]. So far, it is still unclear whether chlorophyllin needs to enter the cells to develop cytotoxic effects, but it is very likely that proximity of chlorophyllin to the cells is important (e.g., to bind to the cell membrane). Microscopic images indicate that chlorophyllin (red fluorescence) is accumulated inside or next to the bacteria (Figure 7A,B). Further studies will be employed to elucidate chlorophyllin’s mechanism of action, both for the light-dependent and the light-independent effects.

One great advantage of chlorophyllin is the approval for food coloring (EU license number E140), reflecting the harmlessness of this substance for humans. Consequently, chlorophyllin-based photosensitization has already been recommended for food decontamination [77,82,85,86]. Scientists still must spend some effort to overcome the outer membrane of Gram-negatives but safety and high capacity in bacteria inactivation make formulations with chlorophyllin (and possibly chlorophyll) a very promising remedy also against bacterial infections of the body surface. Already in 1945, Smith et al. found that chlorophyllin supported healing of wounds, which were infected with *Staphylococcus pyogenes* C-209 in Guinea pigs [87]. Our finding that chlorophyllin killed the Gram-positive *B. subtilis* also in a light-independent way could open new possibilities, e.g., leading to an application against inner infections. In darkness we found that *B. subtilis* cells were killed at chlorophyllin concentrations of 2.5 mg/L. In the pre-antibiotics era Gruskin [88] described successful treatment of persons suffering from *Streptococcus septicemia*, if 250 mL of a 2 g/L chlorophyllin solution was injected every day (500 mg per day) for several subsequent days. For treatment of pancreatitis Yoshida et al. injected a daily dose of 5–20 mg of chlorophyllin per day for 1–2 weeks [89]. As chlorophyll is believed not to exert significant toxicity (if strong light is avoided) it is possible that the acceptable dose for humans is higher than that for bacteria.

Because there is more than one uptake pathway for heme in most pathogens, the development of resistance mechanisms against porphyrins or porphyrin-antibiotic conjugates is―in contrast to other photosensitizers such as methylene blue [90]―highly unlikely [91,92]. Also, a combination of chlorophyllin-application with variations of physical parameters (such as temperature, light intensity or wavelength) may increase killing rate of bacteria. Buchovec et al. [62] described that pulses of strong UV (peak 260 nm, 0,29 J/cm^2^) along with blue light (405 nm, 46.1 J/cm^2^) are more than 10,000-fold more effective than blue light alone to kill *S. enterica* in the presence of 1.5 × 10^−5^ M chlorophyllin.

## 5. Conclusions

Photodynamic treatment using chlorophyll seems a very promising approach. So far, no bacterial resistance mechanism against porphyrins is known, and it is unlikely that such a mechanism will develop in near future. The harmlessness of chlorophyllin allows universal application. We could show that chlorophyllin in combination with light is very effective to control Gram-positive as well as Gram-negative bacteria, but the latter to a lower extent since the outer membrane of Gram-negatives impedes chlorophyllin uptake. Additionally, we could demonstrate that chlorophyllin is more than a photosensitizer killing bacteria such as *B. subtilis* or *E. coli* NR698 in darkness. This suggests a second mechanism of action, which still needs to be elucidated by further studies.

## Figures and Tables

**Table 1:** Approaches for antimicrobial photodynamic therapies.

Photosensitizer	Bacteria	Gram	Results	Ref.
**Riboflavin,** **FLASH-01a,** **FLASH-07a**	MRSA	+	FLASH-01a and -07a destroyed bacteria very effectively; no effect on human keratinocytes	[41]
	EHEC	−		
	*Pseudomonas aeruginosa*	−		
	*Acinetobacter baumannii*	−		
**Hyperforin**	PRSA/MRSA	+	Growth inhibition in all Gram-positive bacteria, but not Gram-negative bacteria and *Candida albicans*	[42]
	*Enterococcus faecalis*	+		
	*Corynebacterium diphtheriae*	+		
	*Pseudomonas aeruginosa*	−		
**Toluidine blue O** + blue LED light; **Porphyrin** + red LED light	*Prevotella melaninogenica*	−	Reduced survival rate	[43]
	*Porphyromonas gingivalis*	−		
	*Aggregatibacter actinomycetemcomitans*	−		
**Phenalen-1-one** derivatives (e.g., SAPYR)	*S. aureus*, MRSA	+	Pronounced antimicrobial efficacy of different phenalen-1-one derivatives	[44]
	*Escherichia coli*	−		
	*Pseudomonas aeruginosa*	−		
	{*Enterococcus faecalis*	+	SAPYR is effective against in vitro biofilms in light	[38]
	*Actinomycesnaes lundii*}	+		
	{*Actinomyces naeslundii*	+	SAPYR exhibits neither uptake nor strong attachment toward bacteria	[39]
	*Fusobacterium nucleatum*	−		
	*Porphyromonas gingivalis*}	−		
**Cationic porphyrins**	*Escherichia coli*	−	Inactivation of localized cells	[45]
	*Pseudomonas syringe*	−	In vitro: strong effect after about 15 min of irradiation (≥10^7^-fold cfu decrease); Ex vivo: about 10^3^-fold cfu decrease	[46]
meso-substituted cationic porphyrins	*Escherichia coli*	−	Chemical composition of external structures seems to have stronger effect on aPDT efficacy than complexity and the number of layers of the bacterial coating.	[47]
	*Aeromonas salmonicida*	−		
	*Aeromonas hydrophila*	−		
	*Rhodopirellula* sp.	−		
	*Staphylococcus aureus*	+		
	*Truepera radiovictrix*	+		
	*Deinococcus geothermalis*	+		
	*Deinococcus radiodurans*	+		
**Hematoporphyrin**, **Chlorophyll a** encapsulated in DOTAP, DPPC, or DMPC vesicles	MRSA	+	Encapsulated chlorophyll has no significant effect of bacteria development (in contrast to free chlorophyll)	[48]
**Chlorin e6**	*Staphylococcus aureus*	+	Photoinactivation,	[49]
			decreased biofilm formation ability	[50]
	*Pseudomonas aeruginosa*	−	Photoinactivation	
	*Escherichia coli*	−	Minor effect	
	*Salmonella enterica* sv. Typhimurium	−	Minor effect	
**Chlorophyllin**	*Listeria monocytogenes*	+	Photoinactivation	[51]
	*Bacillus cereus*	+	Photoinactivation	[52]
	*Escherichia coli*	−	Photoinactivation in presence of ZnO nanoparticles	[53]

cfu: colony-forming unit(s); DOTAP: N-[1-(2,3-dioleoyloxy)propyl]-N,N,N-trimethylammonium chloride; DMPC: L-α-dimiristoyl-phosphatidyl-choline; DPPC: DL-α-dipalmitoyl-phosphatidyl-choline; EHEC: enterohemorrhagic *E. coli*; LED: light-emitting diode; MRSA: methicillin-resistant *S. aureus*; PRSA: penicillin-resistant *S. aureus*; {…} biofilm.

**Table 2:** MIC values of chlorophyllin extracted from spinach against Gram-negative and Gram-positive model strains. The exposure time to chlorophyllin was 24 h at 37 °C in LB. MICs were determined in light (total light dose: ~1000 J/cm^2^, bright columns) or darkness (grey columns).

Titer [cfu/mL]	*Escherichia coli* DH5α		*Bacillus subtilis* 168		*Escherichia coli* NR698	
	Light	Dark	Light	Dark	Light	Dark
1 × 10^8^	>25 mg/L	>25 mg/L	0.5 mg/L	5 mg/L	1 mg/L	>25 mg/L
1 × 10^7^	>25 mg/L	>25 mg/L	0.5 mg/L	2.5 mg/L	0.5 mg/L	>25 mg/L
1 × 10^6^	0.5–1 mg/L	>25 mg/L	0.5 mg/L	2.5 mg/L	0.5 mg/L	5 mg/L
1 × 10^5^	≤0.1 mg/L	>25 mg/L	≤0.1 mg/L	2.5 mg/L	≤0.1 mg/L	2.5 mg/L
1 × 10^4^	≤0.1 mg/L	>25 mg/L	≤0.1 mg/L	2.5 mg/L	≤0.1 mg/L	2.5 mg/L
1 × 10^3^	≤0.1 mg/L	>25 mg/L	≤0.1 mg/L	2.5 mg/L	≤0.1 mg/L	2.5 mg/L
1 × 10^2^	≤0.1 mg/L	>25 mg/L	≤0.1 mg/L	2.5 mg/L	≤0.1 mg/L	2.5 mg/L

**Table 3:** MIC values of chlorophyllin converted from commercial chlorophyll, determined in light (bright columns) or darkness (grey columns).

Titer [cfu/mL]	*Escherichia coli* DH5α		*Bacillus subtilis* 168	
	Light	Dark	Light	Dark
1 × 10^8^	7.5 mg/L	12.5 mg/L	<0.1 mg/L	2.5 mg/L
1 × 10^6^	5 mg/L	12.5 mg/L	<0.1 mg/L	2.5 mg/L
1 × 10^5^	5 mg/L	12.5 mg/L	<0.1 mg/L	2.5 mg/L

**Table 4:** EC_50_ values for chlorophyllin in mg/L, determined in light (bright columns) or darkness (grey columns).

Exposure [h]	*Escherichia coli* DH5α		*Bacillus subtilis* 168		*Escherichia coli* NR698	
	Light	Dark	Light	Dark	Light	Dark
1	n.d.	n.d.	0.00	2.68	0,01	2.21
2	n.d.	n.d.	0.00	1.74	0.09	1.25
3	n.d.	n.d.	0.01	0.82	0.01	0.71
24	n.d.	n.d.	0.29	0.99	0.25	n.d.

n.d.: EC_50_ could not be determined.

**Table 5:** Temperature-dependent MIC values of extracted chlorophyllin against *Bacillus subtilis* 168. MICs were determined in light (bright column) or darkness (grey column).

Temp.	Titer [cfu/mL]	*Bacillus subtilis* 168			
		Light		Dark	
28 °C	1 × 10^8^	▼	≤0.1 mg/L	▼	2.5 mg/L
	1 × 10^6^	▼	≤0.1 mg/L		2.5 mg/L
	1 × 10^5^		≤0.1 mg/L		2.5 mg/L
37 °C	1 × 10^8^		0.5 mg/L		5 mg/L
	1 × 10^6^		0.5 mg/L		2.5 mg/L
	1 × 10^5^		≤0.1 mg/L		2.5 mg/L
42 °C	1 × 10^8^		0.5 mg/L	▲	12.5 mg/L
	1 × 10^6^	▼	≤0.1 mg/L	▲	7.5 mg/L
	1 × 10^5^		≤0.1 mg/L	▲	5 mg/L

▼ decrease, ▲ increase compared to 37 °C.

## Supplementary Materials

The following are available online at https://www.mdpi.com/2076-2607/7/2/59/s1, Figure S1: LB agar plates for the evaluation of CFU ability after incubation to chlorophyllin.
