# Supplementary material for: What an Escherichia coli Mutant Can Teach Us About the Antibacterial Effect of Chlorophyllin
Source: Microorganisms. 2019 Feb 22;7(2):59. doi: 10.3390/microorganisms7020059 (PMC6406390; doi:10.3390/microorganisms7020059)
Supplement: Supplementary file 1 [file microorganisms-07-00059-s001.pdf]

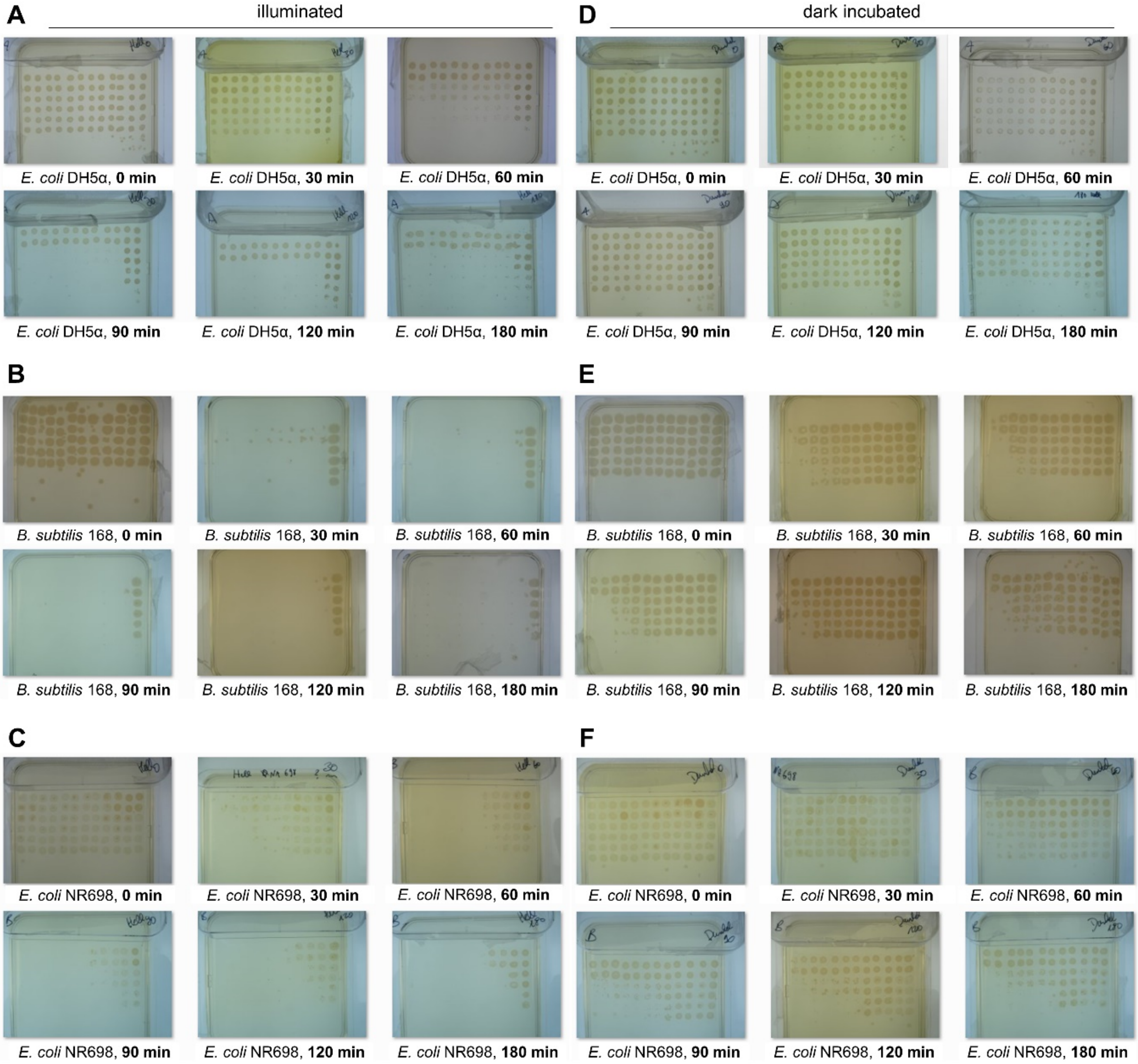

Figure S1. LB-agar plates for the evaluation of the CFU ability after incubation to chlorophyllin. Differently dense liquid cultures of (A) *Escherichia coli* DH5α, (B) *Bacillus subtilis* 168, and (C) *Escherichia coli* NR698 were supplemented with different chlorophyllin concentrations between 0.1 and 25 mg/L. Cells grew in 96-well matrix plates either illuminated with 12 mW/cm^2 (A-C) or protected from light (D-F). Samples (2.5 µL) were drawn at different time points and transferred onto LB-agar plates. After overnight incubation at 37°C in the dark, colony growth was analyzed.
